# A Novel 3D Scaffold for Cell Growth to Assess Electroporation Efficacy

**DOI:** 10.3390/cells8111470

**Published:** 2019-11-19

**Authors:** Monica Dettin, Elisabetta Sieni, Annj Zamuner, Ramona Marino, Paolo Sgarbossa, Maria Lucibello, Anna Lisa Tosi, Flavio Keller, Luca Giovanni Campana, Emanuela Signori

**Affiliations:** 1 Department of Industrial Engineering, University of Padova, 35131 Padova, Italy; annj.zamuner@unipd.it (A.Z.); paolo.sgarbossa@unipd.it (P.S.); 2 Department of Theoretical and Applied Sciences, University of Insubria, 21100 Varese, Italy; 3 Campus Bio-Medico University of Rome, 00128 Roma, Italy; R.Marino@unicampus.it (R.M.); F.Keller@unicampus.it (F.K.); emanuela.signori@ift.cnr.it (E.S.); 4 CNR-Institute of Translational Pharmacology, 00133 Roma, Italy; maria.lucibello@ift.cnr.it; 5 Veneto Institute of Oncology IOV-IRCSS, 35128 Padua, Italy; annalisa.tosi@iov.veneto.it; 6 Department of Surgical Oncological and Gastroenterological Sciences DISCOG, University of Padova, 35124 Padova, Italy; luca.giovanni.campana@gmail.com

**Keywords:** ECM, scaffold, electroporation, breast cancer, hyaluronic acid, self-assembling peptides

## Abstract

Tumor electroporation (EP) refers to the permeabilization of the cell membrane by means of short electric pulses thus allowing the potentiation of chemotherapeutic drugs. Standard plate adhesion 2D cell cultures can simulate the in vivo environment only partially due to lack of cell–cell interaction and extracellular matrix (ECM). In this study, we assessed a novel 3D scaffold for cell cultures based on hyaluronic acid and ionic-complementary self-assembling peptides (SAPs), by studying the growth patterns of two different breast carcinoma cell lines (HCC1569 and MDA-MB231). This 3D scaffold modulates cell shape and induces extracellular matrix deposit around cells. In the MDA-MB 231 cell line, it allows three-dimensional growth of structures known as spheroids, while in HCC1569 it achieves a cell organization similar to that observed in vivo. Interestingly, we were able to visualize the electroporation effect on the cells seeded in the new scaffold by means of standard propidium iodide assay and fluorescence microscopy. Thanks to the presence of cell–cell and cell–ECM interactions, the new 3D scaffold may represent a more reliable support for EP studies than 2D cancer cell cultures and may be used to test new EP-delivered drugs and novel EP protocols.

## 1. Introduction

Cell membrane electroporation (EP) is a physical phenomenon exploited in different emerging cancer treatments such as electrochemotherapy (ECT) and irreversible EP (IRE). Through the administration of properly tuned electric pulses, EP leads to reversible (as in ECT) or irreversible (as in IRE) cell membrane permeabilization. While in ECT, EP increases the uptake and activity of concomitant chemotherapy, in IRE it is applied as a standalone therapy which provokes irrecoverable cell imbalance [1,2,3,4,5,6]. ECT has been first standardized in 2006 and the currently adopted protocols in clinical practice include pre-determined standardized parameters [1,7]. For instance, the electroporation protocol for skin tumors is standardized in terms of voltage and the number of pulses in the framework of the European Standard Operating Procedure on Electrochemotherapy (ESOPE). The voltage amplitude is related to the electrode geometry and in particular to the distance between electrode needles.

Nevertheless, it is well known that the EP conditions have to be tuned when applied to different tumoral tissues [8,9].

With the aim to improve EP-based treatments, efforts are in place to customize pulse protocols and identify the most suitable electric field intensity according to different tumor histotypes.

Until now, the selection of parameters for cell EP has been determined by using in vitro tests on cell suspensions and 2D cell cultures [10,11,12,13,14]. In the first approach, the determination of the electroporation threshold depends on the conductivity of the cell suspension [10,11]. Anyhow, both cell suspensions and 2D cell cultures cannot reliably simulate in vivo tumoral tissues, lacking cell–extracellular matrix (ECM) interactions or, in case of 2D culture, cell–cell interactions in a 3D environment. To overcome these problems, tumor spheroids and hydrogels have been proposed with the aim of reproducing the complexity of tumor tissues in EP studies [15,16,17,18,19,20,21,22]. These models are easy to handle, but present limitations. On one hand, not all cell lines can form spheroids, on the other hand, tumor sensitivity to electric fields is dependent on spheroid diameter [23,24,25,26]. Finally, spheroids are typical tumoral structures but they don’t represent the whole tumoral tissue and do not include cell–ECM interactions [15,25].

Recently, Ivey and Campbell underlined the importance of more reliable 3D tumor tissue models [27,28,29].

In this study, we present a new collagen-free 3D scaffold for cell culture aimed at reproducing as close as possible the tissue organization commonly found in tumors in vivo [30,31]. The new scaffold is a crosslinked and lyophilized matrix based on hyaluronic acid and ionic-complementary self-assembling peptides (SAPs) condensed with the IKVAV (Ile-Lys-Val-Ala-Val) Laminin adhesion motif [32,33,34]. The proposed collagen-free scaffold promotes HCC1569 and MDA-MB231 growth and induces cellular production of ECM. The tumor cell-spheroids, obtained from cell culture in the 3D scaffold, were used in electroporation assays, to test protocols efficiency and to validate the model for future drug delivery studies based on electrochemotherapy.

The aim of the proposed model is related to finding a new in vitro model where cells are immersed in an environment closer to real tissue since they could interact with other cells and with ECM. This could be relevant in EP tests since it is well known that the conductivity of the electroporation buffer influences the cell membrane electroporation [35,36,37]. The presented model would mimic the stroma of the breast cancer tumors in order to improve the efficacy of the in vitro test of electroporation by considering a more complex structure. In fact, it is well known that tissue inhomogeneity could influence the electric field distribution [37,38,39,40]. Currently, the classical tests that use cell suspension to evaluate electroporation set-up lack the inhomogeneity due to the ECM [10,41], even if in recent experiments spheroids were introduced. Spheroids were able to create hypoxic conditions due to cells aggregation in relatively dense space due to uncontrolled proliferation [17,18,25], but they did not include the ECM effect. This paper presents a first evaluation of the use of a new 3D scaffold for cell cultures to be used in electroporation experiments. This new model introduces both cell–cell and cell–ECM interactions, possibly representing a more realistic model of the tumor environment.

## 2. Materials and Methods

### 2.1. Materials for Scaffold Preparation

Hyaluronic acid (MW = 100–1250 kDa) was obtained from Contipro Biotech s.r.o (Dolni Dobrouc, Czech Republic). 1-Ethyl-3-(3-dimethylaminopropyl)carbodiimide (EDC) and Triethoxysilane (TES) from Sigma Aldrich (Steinheim, Germany), and ethanol from VWR Chemicals Prolab (Fontenay-sous-Bois, France). The Rink Amide MBHA resin and the 9-fluorenylmethoxycarbonyl (Fmoc) protected amino acids were purchased from Novabiochem (Merck KGaA, Darmstadt, Germany). The coupling reagents 2-(1*H*-Benzotriazole-1-yl)-1,1,3,3-tetramethyluronium hexafluorophosphate (HBTU) and 1-Hydroxybenzotriazole (HOBt) from Advanced Biotech (Seveso, MI, Italy). *N*,*N*-diisopropylethylamine (DIEA) and piperidine were purchased from Biosolve (Leenderweg, Valkenswaard, The Netherlands). *N*,*N*-dimethylformamide (DMF), trifluoroacetic acid (TFA), *N*-methyl-2-pyrrolidone (NMP) and dichloromethane (DCM) were from Biosolve (Leenderweg, Valkenswaard, The Netherlands). Acetonitrile and TFA were from Sigma-Aldrich, Saint Louis, MO, USA.

### 2.2. Synthesis of a SAP Functionalized with Laminin Adhesion Sequence

The self-assembling peptide (SAP) used is an analogue of EAK 16 module II [34,42,43] (a SAP with 16 amino acids that alternates pairs of negative (E, glutamic acid) and positive (K, lysine) charges, separated by a hydrophobic amino acid (A, alanine) [44]) with the substitution Ala→Abu (Abu = α-aminobutyric acid, Ala = Alanine) and the introduction of the Laminin sequence IKVAV (Ile-Lys-Val-Ala-Val) at its C-terminal. The peptide was synthesized by Fmoc chemistry using Rink Amide MBHA resin (0.7 mmol/g; scale 0.125 mmol) and the synthesizer Syro I (Multisynthec, Witten, Germany). The first three amino acids and the last sixteen amino acids were introduced through double couplings [45]. At the end of the synthesis, the Fmoc was removed, the resin was washed with DCM and dried for 1 h under vacuum [45]. The peptide was cleaved from the solid support with contemporary side-chain deprotection using the following mixture: 0.125 mL MilliQ water, 0.125 mL TES, and 4.750 mL TFA over 90 min, under magnetic stirring. The resin was filtered, and the reaction mixture was concentrated. The crude peptide was precipitated with cold diethyl ether. The peptide was purified by Reverse Phase-High Performance Liquid Chromatography and its identity was ascertained by MALDI-TOF (Matrix Assisted Laser Desorption Ionization–Time of Flight) mass spectrometry (theoretical value = 2239 Da; experimental value = 2236.32 Da)

### 2.3. Preparation of the 3D Scaffold

**Figure 1:**
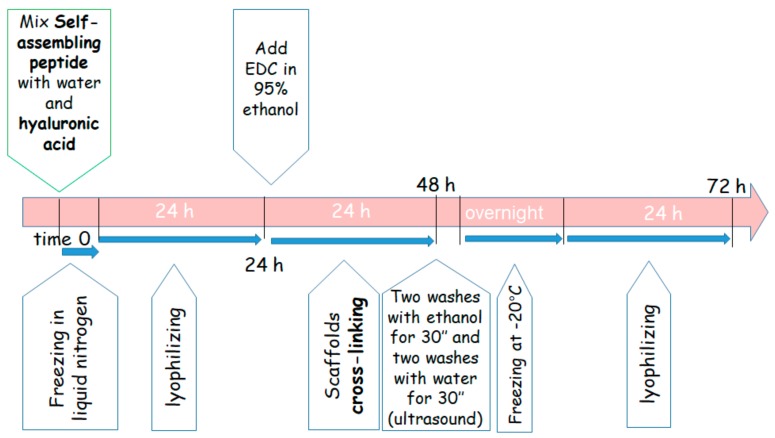
Steps of the procedure for the 3D scaffold preparation.

SAP (4.2 mg, 0.12% *w*/*v*) was dissolved in 3.5 mL of MilliQ water under stirring. Hyaluronic acid (108 mg, 3% *w*/*v*) was slowly added to the solution. The dense solution was divided into the 5 wells of a chamber slide, frozen in liquid nitrogen and lyophilized. The scaffolds (dimension: 8 × 10 × 5 mm) were cross-linked through reaction with 50 mM EDC in 95% ethanol for 24 h. The scaffolds were washed in an ultrasound bath twice with ethanol for 30 s and twice with MilliQ water for 30 s. Finally, the scaffolds were frozen at −20 °C and lyophilized. The 4 days long procedure is illustrated in Figure 1.

### 2.4. Cell Culture in the 3D Scaffold

The MDA-MB231 and HCC1569 cells lines were purchased at ATTC (www.atcc.org) and cultured in flasks, in culture media (DMEM (Corning, Mediatech Inc., Corning, NY, USA) added with 1% Penicillin/streptomycin (Gibco, Invitrogen, Waltham, MA, USA), 1% l-Glutamine (Sigma-Aldrich, St. Louis, MO, USA), and 10% fetal bovine serum (FBS, Gibco, Invitrogen, USA) for MDA-MB 231 and with 10% FBS North America for HCC1569, respectively) before seeding them in the 3D culture environment.

For both the histological analysis and electroporation experiments, cells were detached with 0.25% *w*/*v* trypsin/0.53 mM EDTA solution (Corning), centrifuged (1200 rpm, 5 min), and re-suspended in the proper culture medium. Cells were counted and seeded inside the 3D scaffold (3 × 10^5^/cm^2^ in 500 µL of medium) hydrated for 1 h before the seeding in ultra-low attachment 24 wells plates. Finally, scaffolds with cells were cultured at 37 °C for 24 h, 3 days, or 7 days prior analysis. The electroporated cultures were incubated for 24 h or 7 days, whereas for the histological analysis also a 3 days old sample was prepared. All samples were tested in triplicate.

### 2.5. Culture Analysis

#### 2.5.1. Viability

Cell viability was evaluated using the trypan blue assay and propidium iodide assay. The trypan blue solution (0.4% in water) was added to the cell culture. A drop of the cell culture was deposed on a microscopy glass, covered with a coverslip and observed with a light microscope Leitz DMRB (Leica Microsystems Wetzlar GmbH, Wetzlar, Germany) at 20× magnification. Images were acquired with a digital Nikon DSU-1 camera (Amsterdam, Netherlands). The Propidium iodide assay (100 µg/mL in deionized water) was added to the culture medium just before the analysis at inverted microscope (excitation wavelength = 620 nm). The Trypan blue and propidium iodide assays were performed at different time points (24 h, 3 days and 7 days).

#### 2.5.2. Fluorescent Staining

The 3D cell culture was stained with Hoechst 33342 (ThermoFisher, Waltham, MA, USA), Acridine Orange (ThermoFisher, Waltham, MA, USA) and Propidium Iodide (Sigma Aldrich, Saint Louis, MO, USA) solutions in order to dye the nucleus, identify alive and apoptotic cells, identify the necrotic cells, respectively. The nucleus of the cell is in blue due to Hoechst 33342 (5 µg/mL in PBS) [46,47]. Propidium Iodide (100 µg/mL in deionized water) dies selectively in red the nucleus of necrotic cells and is discarded by alive or apoptotic cells [48,49,50], while Acridine Orange (10 µg/mL in deionized water) dies the cell cytoplasm and nucleus in green if the cells are alive [46,47,51]. In acridine orange staining, apoptotic cells are characterized by disaggregated DNA, while alive cells possess a well distinguishable nucleus [52,53,54,55].

After staining, the 3D cultures were observed with an inverted microscope (Nikon Eclipse Ti, Amsterdam, Netherlands) at 20× magnification acquiring bright-field images and fluorescence images using DAPI, FITC, and TRITC filter sets. All the images were acquired separately with a camera (ANDOR, Neo sCMOS, Nikon, Amsterdam, Netherlands) and superposed using ImageJ software [56,57]. From the images, the cell size was determined, and alive and necrotic cells were evidenced.

### 2.6. Morphological Analysis

#### 2.6.1. SEM Analysis 

The scaffold was observed at the Scanning Electron Microscope (SEM Cambridge Stereoscan 440 SEM, Cambridge, UK). The freeze-dried scaffolds were sputter-coated with gold (EMITECHK950x Turbo Evaporator, EBSciences, East Granby, CT, USA) and images at 500× magnification were acquired as in [58].

The scaffold was analyzed also at the Environmental Scanning Electron Microscope (ESEM Quanta 200, manufactured by FEI, Hillsboro, OR, USA) without any metallization.

#### 2.6.2. Inclusion for Histological Evaluation

The 3D cell cultures were included in 2% agarose gel (Lonza Seakem, LE agarose cod.n.50004) just 1 h after the end of the experiment to obtain a macroscopic block suitable for slicing. After agarose inclusion, the samples were fixed by cooling at −20 °C and then sliced using a cryomicrotome (Leica CM 1850). The slices were deposed on a microscopy glass and properly stained. A sample of the matrix without cells was treated in the same way for comparison.

#### 2.6.3. Staining

The sections of 3D culture samples were stained with Hematoxylin and Eosin (H&E) (Biooptica, Harris Hematoxylin, cod. n. 05-M06004; Eosin G aqueous solution 1% cod. n. 05-10002-L, Milan, Italy), Masson trichrome (Biooptica, cod. n. 04-010802 or DIAPATH cod. n. 010224, Milan, Italy), and Weigert Van Gieson methods (Biooptica, cod. n. 04-051802, Milan, Italy). The H&E stain is a generic staining for cells and extracellular matrix. Instead, the Masson trichrome and Weigert Van Gieson are specific stains for collagen and connective tissues. The first method stains the extracellular matrix in red and the cells in a red-brown color, while the second dies in blue or in green the collagen fibers, in dark the cells and in red other extra cellular matrix components. The latter stain method shows the cells in black, collagen in red and the connective component in yellow. The sample without cells was stained only with H&E for comparison.

#### 2.6.4. Imaging of Sliced Samples

Each sample was observed using the microscope Leitz DMRB at 20× magnification. Each image was acquired with a digital Nikon DSU-1 camera.

### 2.7. Electroporation

#### 2.7.1. 2D Cell Culture vs. 3D Cell Culture Electroporation

The 2D cell culture was used as a comparison in the electroporation tests. In particular, the MDA-MB231 cells used in 2D cell cultures for the evaluation of the proper electroporation threshold in terms of applied voltage were prepared as 3D cell cultures and seeded in 24 wells plates (5 × 10^4^/cm^2^) treated for cell adhesion. They were seeded in DMEM culture medium added of FBS 10% *v*/*v*, 1% Penicillin/streptomycin, and 1% l-Glutamine for 24 h before the electroporation treatment. The 24 wells plates were incubated at 37 °C and 5% CO_2_. The experiment was repeated 2 times applying 6 different voltages, and then 12 wells were seeded.

#### 2.7.2. Pulse Protocol

The set-up of the pulse protocol was performed on 2D cells, cultured in monolayer 24 h before the pulse application. Before electroporation, the medium was substituted with the electroporation buffer as reported in [59,60,61]. The electroporation buffer (pH 7.4) contained 10 mM K_2_HPO_4_/KH_2_PO_4_, 1 mM MgCl_2_, 250 mM sucrose, and 30 µM propidium iodide (PI) (Sigma, St. Louis, MO, USA). Propidium iodide is a fluorescent dye which permeates the electroporated cells, but it is generally excluded from viable ones [62].

**Figure 2:**
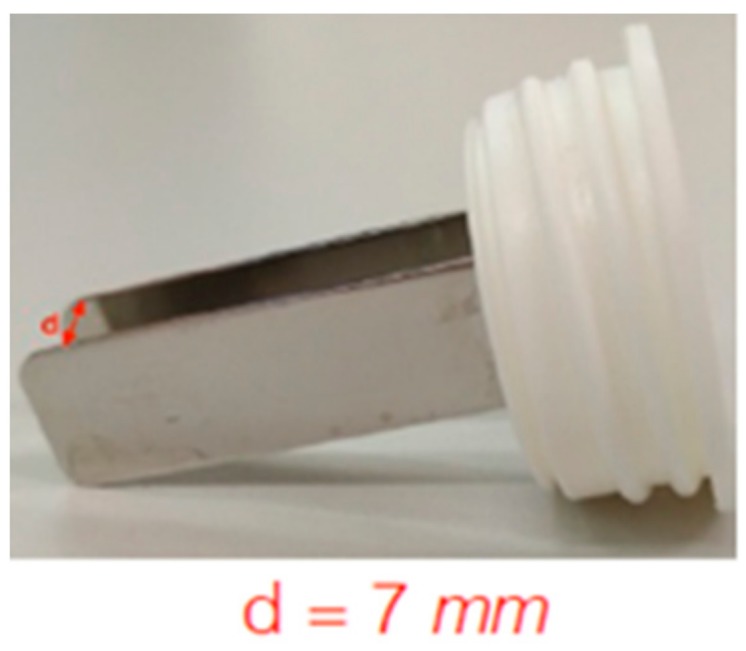
The electrode used in electroporation.

The voltage amplitude to achieve EP in at least 90% of cells was determined by applying a sequence of 8 voltage pulses, 100 µs long at 5 kHz, at different amplitude to each well through a plate electrode. The electrode was formed by two 3 cm-long, 1 cm-large stainless-steel plates, with a gap of 7 mm (Figure 2). The voltage pulses were applied by a pulse generator EPS-02 (Igea, Carpi (MO), Italy). The electrode was immersed in the electroporation buffer to the well bottom. The applied voltages ranged between 0 V and 800 V (0, 200, 300, 400, 600, 700, and 800 V) with an applied electric field between 0 V/cm and 1143 V/cm (0, 143, 429, 571, 857, 1000, and 1143 V/cm). These experiments were carried out to determine the voltage to be applied to the 3D scaffold in order to achieve effective electroporation.

#### 2.7.3. 3D Cell Culture Electroporation

500 µL of medium were used for 3D cell culture. Before electroporation, half of the medium was discarded and substituted with the electroporation buffer containing 60 µM of the fluorescent dyes PI. The 3D cell culture electroporation was performed using the same plate electrode and applying 8 voltage pulses, 100 µs long, at 5 kHz with an amplitude of 680 V (electric field of 1000 V/cm). The used voltage was selected through the 2D culture experiment and corresponds to the voltage applied to the electrode (Figure 2) able to assure electroporation to the maximum number of cells (maximum red intensity).

#### 2.7.4. Evaluation of Electroporation

The occurrence of electroporation was evaluated by adding PI immediately before the delivery of the voltage pulses. The growth medium was replaced with the electroporation buffer (pH 7.4, 10 mM K_2_HPO_4_/KH_2_PO_4_, 1 mM MgCl_2_, and 250 mM sucrose) containing 30 µM of the fluorescent dye.

## 3. Results

### 3.1. 3D Scaffold Morphology

**Figure 3:**
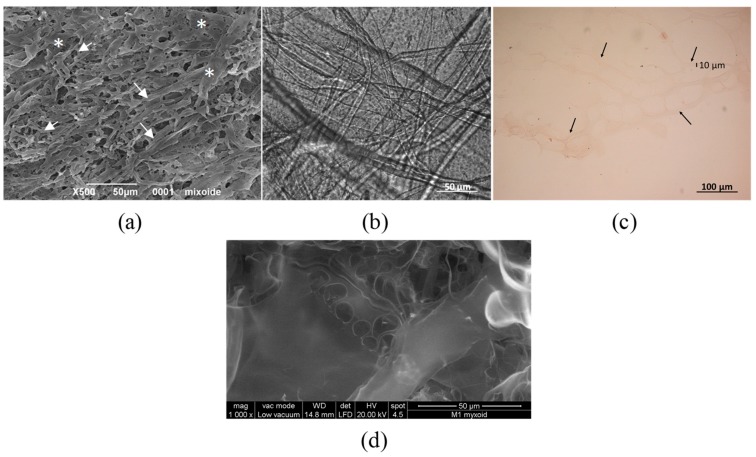
SEM images of the dried scaffold (panel **a**, left) and of the scaffold after hydration with DMEM medium for 2 h of incubation at 37 °C for 7 days with medium (panel **b**), inclusion in agar and staining with H&E (panel **c**, right). Panel **d**, ESEM image of the scaffold at 1000× magnification.

In the SEM image, the 3D scaffold appears like a sponge with large flat areas (white stars in Figure 3a) and elongated structures (white arrows in Figure 3a). The position of these structures is irregular and form a crosslinked network. The 3D scaffold morphology changes with hydration (3 hrs in cell culture medium) as shown in Figure 3b: the sample presents fibers with random directions with a diameter less than 50 µm. Finally, Figure 3c shows the scaffold incubated at 37 °C with the culture medium for 7 days included in agar and stained with H&E. In this case, the sponge structure is evidenced by black arrows and the size of the fiber (10 µm) is reported [58]. The analysis of the dry 3D scaffold was repeated using ESEM instead of SEM to avoid metallization: The image reported in Figure 3d shows a is sponge-like structure with holes and flat surfaces similar to those observed in agar inclusion (Figure 3c).

**Figure 4:**
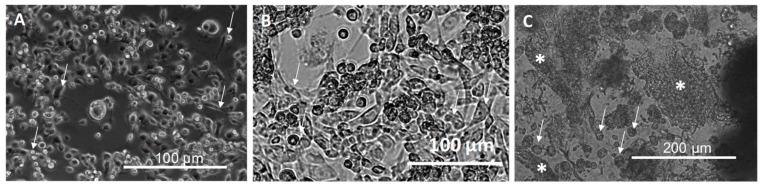
Images acquired in bright field using inverted microscopy of the MDA-MB231 cells cultured in 2D monolayer (10× magnification) observed after (**A**) 24 h and (**B**) 3 days and (**C**) 3D scaffold (20× magnification) and in m. White arrows indicate the cells, whereas white stars in panel (**C**) the extracellular matrix deposed by cells.

The 3D culture of MDA-MB231 cells on the scaffold (Figure 4B) compared with a classical 2D MDA-MB 231 cell culture (Figure 4A) shows some peculiarities, both in cell shape and in the presence of ECM. In both images, the white arrows highlight the cells. In the 3D culture, the cells are more round or polygonal-shaped with respect to the ones present in the 2D culture and cultured in the medium for 24 h and 3 days at 37 °C, where the spreading, induced by the attachment to the plastic bottom, is visible. In the 3D culture, the white stars (Figure 4B) locate the extracellular matrix produced by the cells.

### 3.2. Cellular Viability and 3D Organization

**Figure 5:**
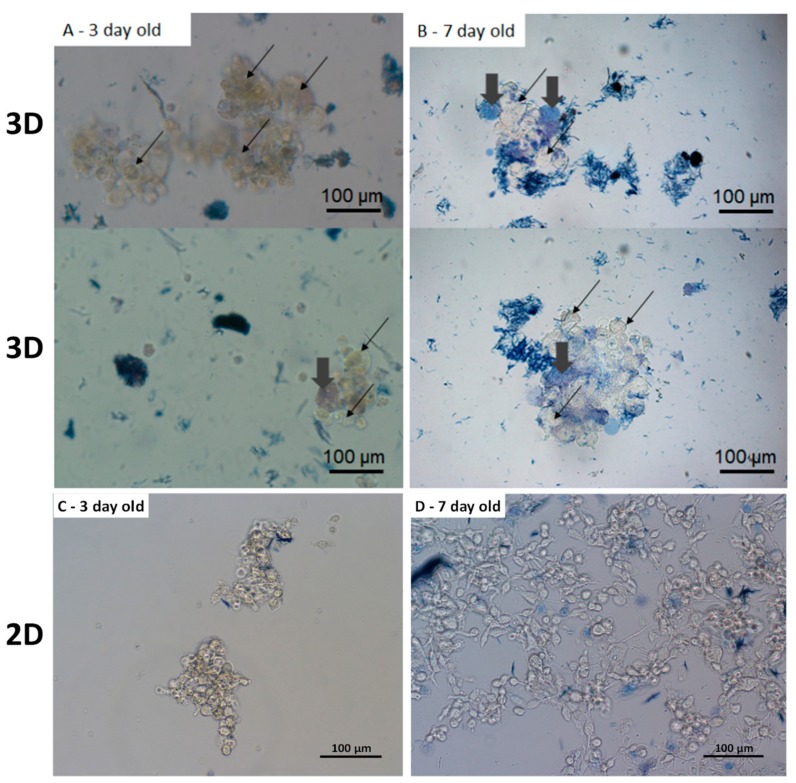
Images acquired in bright field of the cell MDA-MB231 cultured in 3D scaffold and dyed with trypan blue immediately before the observation and posed on a microscopy glass. Images refer to cells cultured for (**A**) 3 days and (**B**) 7 days. Black arrows indicate alive cells, whereas, big grey arrows point on the dead cells. (20× magnification). In the upper images of Figure 5 (**B**) (7 days of cultures) 9 cells among the 42 detected are blue (79% of viability), whereas, 11 cells of the 56 detected in the bottom image are blue (81% of viability). Panel (**C**) and (**D**) show the Trypan blue assay on a 2D cell culture seeded 3 days and 7 days, respectively, before observation (100% of viability).

Trypan blue assay (Figure 5) evidences that a small number of cells died at 3 days (some dead cells are pointed by large grey arrows in Figure 5A). In this image only 5% of cells are blue (dead cells) the others, pointed by the thin black arrows, appear uncoloured (alive cells). After 7 days, the number of dead cells increased up to 18% of the detected cells. In conclusion, in the proposed 3D scaffold the cell viability after 3 and 7 days of culture is similar to that recorded in spheroids [15,22,26]. As comparison, in a 2D culture observed 3 days after seeding the trypan blue assay showed 100% vitality as reported in Figure 5C; wheread at 7 days after seeding the vitality is closer to 95% (Figure 5D).

**Figure 6:**
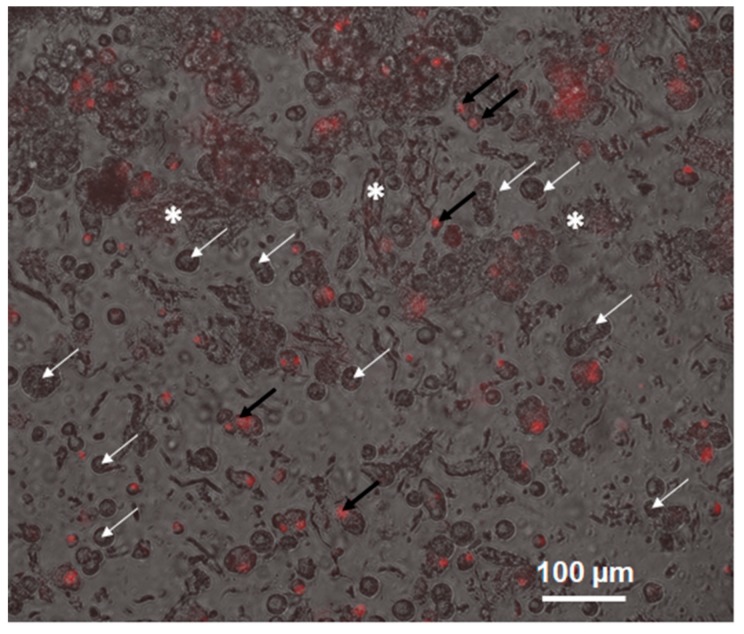
Image in bright field superposed to the corresponding images in red acquired using inverted microscopy of the cell MDA-MB231 cultured in the 3D scaffold (20× magnification). White arrows indicate alive and black arrows dead cells whereas white stars the extracellular matrix deposed by cells. Vitality is 78%.

The Propidium Iodide assay (Figure 6) shows the viability of the cells in the 3D cell culture. The bright-field image was superimposed to the corresponding image of the red fluorescence. The red cells are necrotic (some are pointed by the black arrows), while those able to discard propidium are alive (pointed by the white arrows). In the sample reported in Figure 6, the viability is 78% in agreement with the Trypan blue assay results.

**Figure 7:**
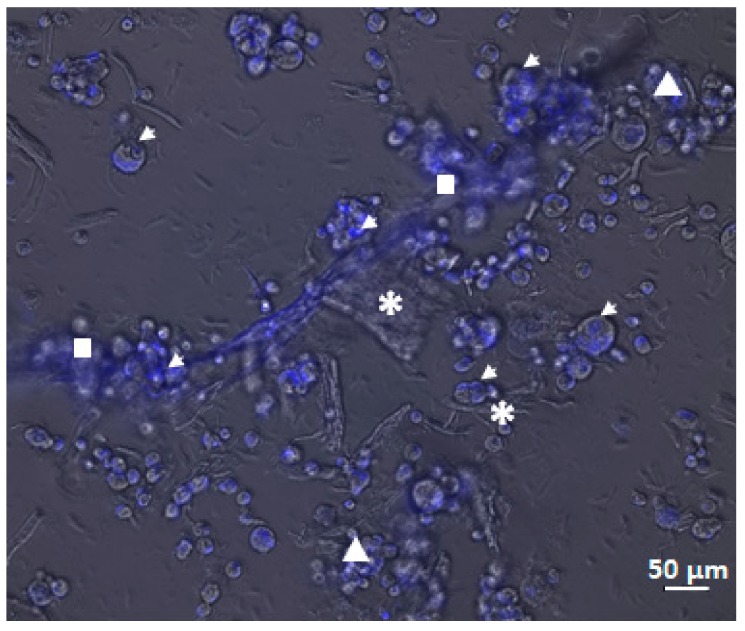
Image of the cell MDA-MB231 cultured in the 3D scaffold at 7 days in bright field superposed to the corresponding images in blue fluorescence (cells dyed with Hoechst 33342) acquired using inverted microscopy (20× magnification). White arrows indicate cells, whereas white stars the extracellular matrix deposed by cells, white triangle, groups of cells and white square, cells bonded to the extracellular matrix (ECM).

**Figure 8:**
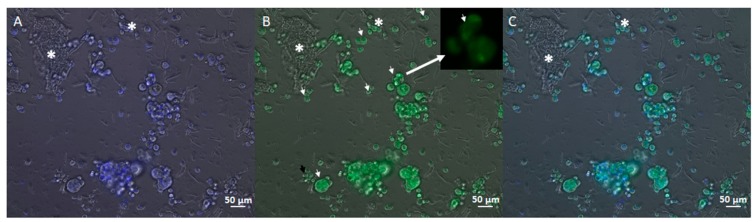
Inverted microscopy images of the cell MDA-MB231 cultured in the 3D scaffold at 7 days (20× magnification). (**A**) Hoechst 33,342 labelled cells; (**B**) Acridine Orange labelled cells; (**C**) superimposition of (**A**,**B**) images. Acridine Orange stains viable cells and labels the DNA in the nucleus in light green. The white stars represent the matrix deposed by the cells. In panel (**B**), is visible a cell in division (black square in the upper part of the images). In this case, DNA separation is visible.

The other fluorescent stains evidence the morphology of the cells. The MDA-MB231 3D culture (Figure 7), stained with the fluorescent dye Hoechst 33342, shows both the cells (marked in blue) and the ECM. In particular, in the centre of the image, the organization and attachment of the cells to the scaffold and to the extracellular matrix components (indicated by the white stars) are evident. The cells appear organized in clusters (white triangles) or in groups bonded to the ECM (white squares). The same organization is shown in Figure 8 thanks to Acridine Orange staining. Also in this image, the ECM is marked with white stars and cells with white arrows. The green cells are living and the superposition between the green image and the blue image indicates that 100% of cultured cells were living at 7 days from seeding.

### 3.3. De Novo Cellular Synthesis of Extracellular Matrix

**Figure 9:**
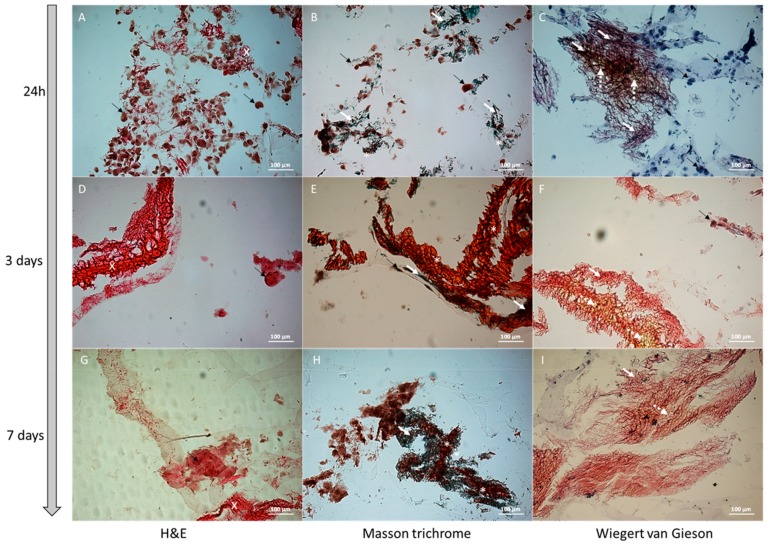
Histological images acquired using inverted microscopy of the cells MDA-MB231 cultured in the 3D scaffold (20× magnification) and stained with H&E, Masson trichrome and Wiegert Van Gieson methods for cultures 24 h, 3 days, and 7 days old. The white arrows indicate collagen stained by Masson trichrome method, black arrows show cells, dotted white arrows highlight the connective stained with Wiegert Van Gieson method, white crosses indicate the extracellular matrix stained by H&E method and the white stars show the extracellular matrix stained in red by Masson trichrome staining.

**Figure 10:**
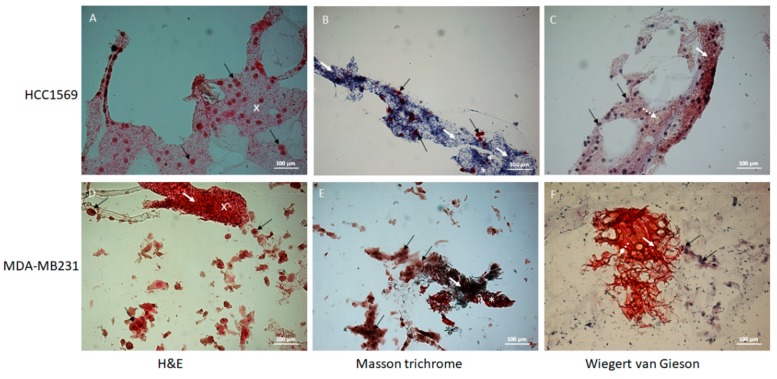
Histological images acquired using inverted microscopy of the cells MDA-MB231 and HCC1569 cultured in the 3D scaffold for 7 days. Images acquired using inverted microscopy of the cell MDA-MB231 cultured in the 3D scaffold (20× magnification) and stained with H&E, Masson trichrome and Wiegert Van Gieson methods. The white arrows indicate collagen stained by Masson trichrome method, black arrows cells, dotted white arrows the connective stained with Wiegert Van Gieson method, white cross the extracellular matrix stained by H&E method and the white stars the extracellular matrix stained in red by Masson trichrome stain. More samples were analyzed at each analyzed times.

The images acquired at the microscope with 20× magnitude of the cells seeded in the 3D scaffold and stained with H&E, Masson trichrome and Weigert Van Gieson methods at different culture times (24 h, 3 days and 7days), are shown in Figure 9 and Figure 10.

Figure 9 shows modifications of MDA-MB231 3D culture cell organization and the increment of the extracellular matrix molecules deposed by the cells in the time frame between 24 h and 7 days. The comparison between the plain scaffold, treated with culture medium for 7 days (Figure 9C), with the cell-seeded scaffold, reported in Figure 9 (panels G,H,I) evidences the appearance of the new ECM deposed by cells. Masson trichrome (MT) and Weigert Van Gieson (WG) staining give information about the nature of de novo synthesized ECM components: collagen (marked in green for MT or in red for WG) and connective components (marked in yellow for WG).

In Figure 9, the cells seeded into the scaffold, after 24 h of culture, appear isolated and present a defined round shape. We suppose this is due to the prevalence of cell-scaffold junctions on cell–cell junctions.

At 24 h the cells are crowded into the semi-transparent 3D scaffold. At increased culture times (3 days and 7 days) the cells show more cell–cell connections and are attached to the scaffold and to the new deposed ECM: the cells appear less distinguishable with respect to the condition registered at 24 h.

In Figure 10 the images of the 3D scaffold seeded with two different cell lines (HCC1569 and MDA-MB231) at 7 days are reported. The HCC1569 cells appear separated, round-shaped and homogenously attached to the scaffold structure, surrounded by abundant ECM. Differently, the MDA-MB231 cells are strictly connected to each other and the ECM appears more dense and predominant in some areas (white arrows and white stars) creating an inhomogeneous distribution between cell clusters and dense ECM areas. In HCC1569 culture, the WG staining shows collagenous fibers (vibrant red color) and rare components of connective tissue (yellow). In MDA-MB231 culture there is a mixture of collagen and connective stroma (green and red colors respectively in MT stain; red and yellow colors respectively in WG stain). The blue or green areas due to MT staining demonstrate that in both cell cultures there is collagen deposition. This result makes our scaffold peculiar and interesting because the presence of collagen in tumor tissue is well documented [63,64].

### 3.4. Application of the New Scaffold: Results of the Electroporation of MDA-MB231 Cell Culture

**Figure 11:**
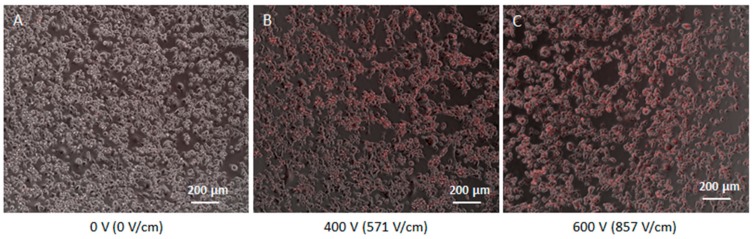
Monolayer cell culture after the treatment with 8 voltage pulses with amplitude (**A**) 0 V (E = 0 V/cm), (**B**) 400 V (E = 571 V/cm) and (**C**) 600 V (E = 857 V/cm). Experiments repeated 2 times.

**Figure 12:**
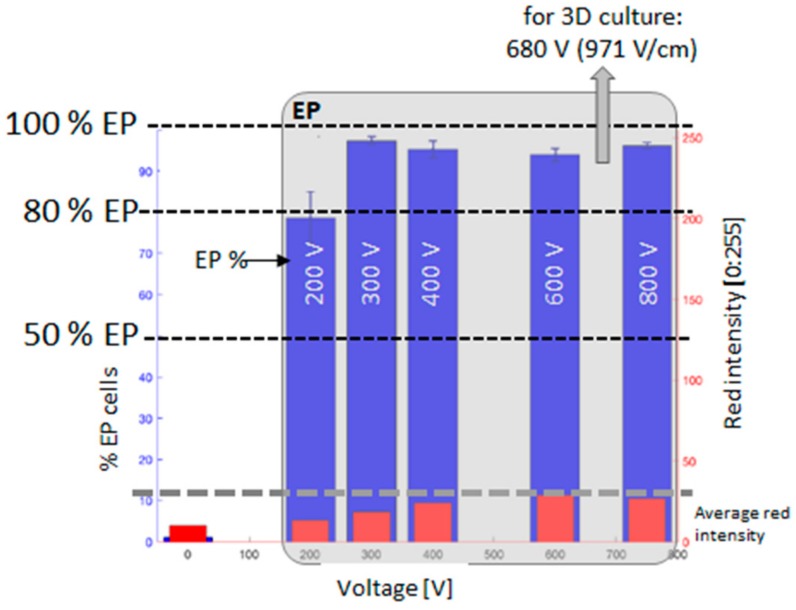
The percentage of electroporated cells and red intensity average as a function of the amplitude of the sequence of 8 voltage pulses.

The electroporation procedure employed voltage pulses to permeabilize the cell membrane. Figure 11 and Figure 12 show the results of the evaluation of the electroporation threshold parameters. This threshold corresponds to the voltage amplitude of a sequence of 8 pulses, 100 µs duration, applied using a plate electrode with plates 7 mm distant able to electroporate a sufficient number of cells. In particular, these electric parameters assured cell electroporation up to 90% of the cells seeded in monolayer. Figure 11 shows the monolayer cell culture after the treatment with 8 voltage pulses at different amplitudes: 0 V (E = 0 V/cm), 400 V (E = 571 V/cm), and 600 V (E = 857 V/cm). The first image represents the negative control with no electroporation, whilst the other two lead to 90% of electroporated cells, as underlined by the two different red intensities. All the experiments were repeated twice. In particular, Figure 12 shows the effect of the voltage increment on the number of electroporated cells, as well as the increase of the red intensity due to Propidium Iodide penetrated in electroporated cells. The percentage of electroporated cells was evaluated counting electroporated and non-electroporated cells (Figure 11). The red intensity average was evaluated on the entire image filtering on red color. In Figure 12 the relative intensity of the voltage chosen for experiments on 3D culture (680 V) is shown. From the reported analysis, the 8 pulses with amplitude 680 V (E = 971 V/cm) are able to electroporate more than 90% of the cells.

**Figure 13:**
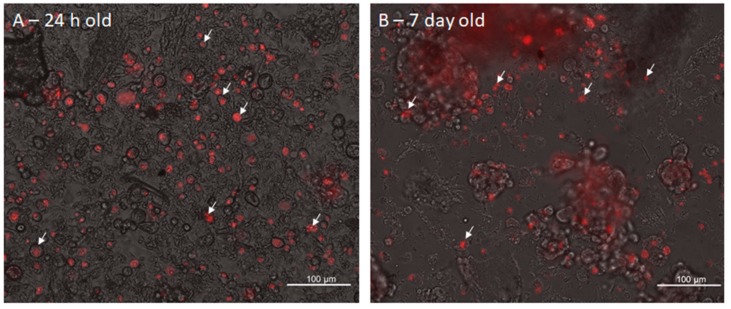
Fluorescence image (Propidium Iodide) of electroporated 3D cultures at 24 h (**A**) and 7 days (**B**). White arrows indicate electroporated cells.

Figure 13 shows two images acquired using the fluorescence microscope, obtained after the electroporation of two 3D cultures at 24 h (panel (A)) and at 7 days (panel (B)).

### 3.5. Comparison of the 3D Culture Histology with Tumor Histology

**Figure 14:**
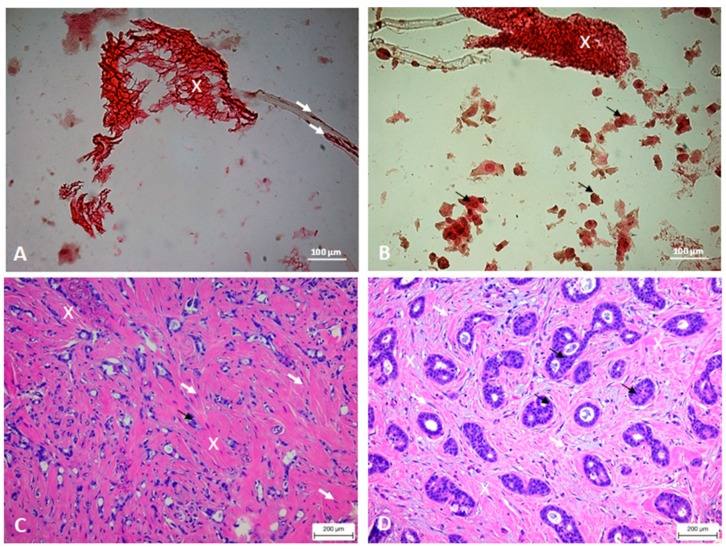
(**A**,**B**) Histological images acquired using inverted microscopy of the cells MDA-MB231 cultured in the 3D scaffold for 7 days (20× magnification) and stained with H&E. (**C**,**D**) histological images of an infiltrating ductal carcinoma of the breast (two different cases). The white crosses indicate collagen stained by H&E method, white arrows the cells.

Histological images acquired using inverted microscopy of the MDA-MB231 cells cultured in the 3D scaffold for 7 days (20× magnification) and stained with H&E (panel A and B in Figure 14) are compared with histological images of an infiltrating ductal breast carcinoma (two different cases, panel C and D in Figure 14). In fact, in this type of tumor tissue the fibrous stroma is well visible (white crosses in Figure 14, panels C and D), which consistency could be both loose or dense [65]. The cells show both elongated and round shape. The white arrows mark elongated cells attached to the 3D scaffold in Figure 14A and elongated cells immersed in the stroma of the real tumor in Figure 14C; whereas the black arrows in Figure 14B,D mark groups of cells with a shape close to round in 3D scaffold culture and real tumor, respectively.

**Figure 15:**
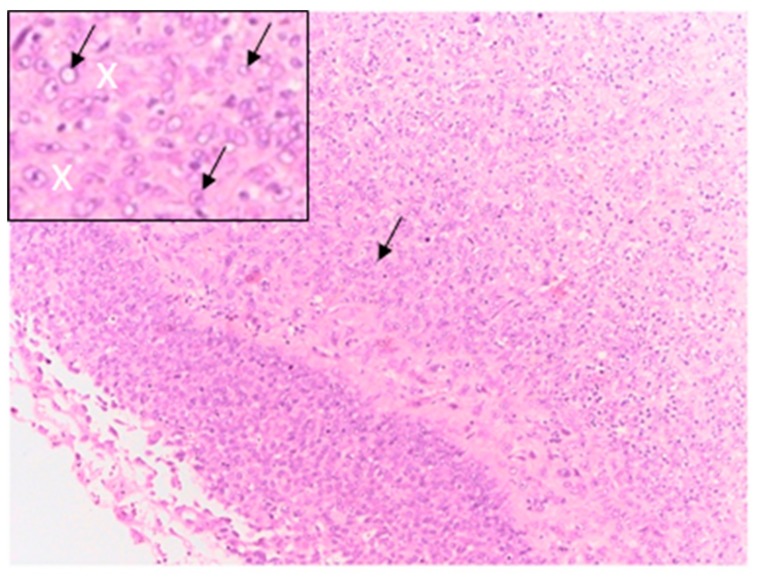
Histological image and zoom of the central region (square in the left corner) of a metaplastic carcinoma stained by H&E method. The white crosses indicate collagen, black arrows the cells.

Instead, Figure 15 reports the image related to a metaplastic breast carcinoma [66,67] that is compared with the 3D culture realized seeding cells from the HCC1569 line. In metaplastic carcinoma the cells are pleomorphic with big nuclei, poor of cytoplasm and shape close to round (black arrows). They are organized in a uniform distribution surrounded by fibrous stroma (white crosses). In this type of tumor sometimes the stroma can be also similar to the one of sarcoma [65].

## 4. Discussion

New and more reliable 3D tumor tissue models are becoming of pivotal importance for drug delivery studies [27,28,29].

In this work we present a new 3D scaffold able to modulate cell shape and to induce an extracellular matrix deposit around cells, thus making this model more useful than 2D cancer cell cultures to test drug delivery protocols by electroporation.

Following the important study of Kenny et al. [68], to validate our scaffold we chose MDA-MB231 and HCC1569 breast cancer cell lines cultured as monolayer and in our 3D model, to compare cells organization, viability, and efficiency of EP protocols. Our results in cells cultured in the proposed 3D scaffold were analyzed up to after 7 days of culture. The results from this experimental work show a first evaluation of the use of a new 3D scaffold for cell cultures suitable to be used in electroporation experiments to test electroporation efficiency in presence of ECM. In fact, the presented model introduces both cell–cell and cell–ECM interactions, that represent a more realistic condition of the tumor environment.

The results of cells cultured in the proposed 3D scaffold, analyzed after 7 days, show different cell morphology with respect to what is reported in the work of Kenny et al., where the same cell lines were cultured in Matrigel [68]. Kelly et al. state that, whereas the HCC1569 and MDA-MB231 lines adopted largely non-distinct morphologies when cultured as monolayers (2D), dramatic differences emerged when grown on a 3D scaffold. We can now add another experimental observation to their claim demonstrating that the composition and the biomechanical properties of the 3D scaffold can determine the morphology of cancer cells.

For instance, in Kenny’s paper [68], HCC1569 cells cultured in Matrigel present a mass-like growth with strong adhesion between adjacent cells, whereas in our scaffold the cells are generally well separated and surrounded by ECM.

Considering MDA-MD231 culture, in [68] the cell organization is classified as ‘stellate’ with elongated cells and a not well definite arrangement. In our scaffold, the cells are organized in not well-defined groups. Moreover, the single cells show a not-well definite shape that in some cases could be approximated as round and in others as elongated. Considering Figure 5, Figure 6, Figure 7 and Figure 8, it is evident that in our 3D scaffold of hyaluronic acid and SAPs the cells are able to aggregate to form masses. In fact, in the MT staining, the green color indicates the presence of collagen, important constituent of ECM. This observation can be reinforced by the presence of red-marked components after WG staining. It is known that collagen component was already evidenced in breast cancer, by using MT staining. In the same study, Hamad et al. [69] reported that some breast tumors show an abundant fibrous component, with collagen constituent, and a poor cell component. The same observation is present in the papers by Insua-Rodriguez and Goddar, where an abundant presence of fibrillary collagen is highlighted in the mammary tumors [70,71]. Furthermore, other EMC components should be evidenced, such as fibronectin, laminin, or proteoglycans. In agreement with these observations, the culture of HCC1569 and MDA-MB231 on our hyaluronate-SAPs scaffold showed other ECM components different from collagen.

These can be considered proofs that the characteristics of the 3D scaffold and the nature of the tumor cells jointly guide the architecture of the tumoral tissue, and that the scaffold of hyaluronic acid and SAPs induces the production of ECM rich in collagen as observed in human mammary tumors. Nonetheless, our final goal was to realize a tumoral tissue model in vitro, to study reversible electroporation process. Consequently, our aim was to approach as much as possible the breast tumor tissues. With this purpose, Figure 14 compares histological images of the 3D scaffold with histological images of an infiltrating ductal breast carcinoma.

The MDA-MB 231 cells, cultured in the 3D scaffold, are immersed in a fibrous stroma marked with white crosses (Figure 14, panel A and B), with some elongated cells attached to the scaffold structure (white arrows in Figure 14A). In the real tumor histology, the cells immersed in the stroma show an elongated shape as indicated by the white arrows in the human tissue image (Figure 14C), while the cells that form the glandular lumen (Figure 14D) have a more round shape, forming islands (black arrows). In the latter case, the cells are also more regular in shape. Also in the 3D culture, some cells with a shape close to round are arranged in groups (black arrows in Figure 14B).

Considering the images related to HCC1569 cell line in Figure 10, the cells of the 3D culture show a round shape with big nuclei and they are poor of cytoplasm (black arrows). They are attached to the scaffold structure and surrounded by the stroma, that is composed in prevalence by collagen fibers, as evidenced by MT and WG staining (white arrows). Therefore, the 3D cell cultures show similarity with the metaplastic tumor (Figure 15) in terms of the presence of the stroma around the cells and the shape of the cells close to round. 

All these results make our 3D scaffold very reliable for in vitro studies on drug delivery based on electroporation to translate in vivo.

For this reason, we moved to experiments to test electroporation conditions efficiency in our 3D scaffold. In this aim, electroporation in the 2D model was performed to set up the electric conditions threshold before the 3D electroporation experiments, to define a reference electric field intensity in known conditions for the cell line used. Once selected the best parameters, our results demonstrate that, in the case of the 24 h 3D culture in Figure 13A, the electroporated cells are close to 85%. Considering the 3D culture at 7 days in Figure 13B, a percentage of red cells higher than 80% is again visible in the aggregates and inside the ECM, as shown in the upper part of the figure. These percentages of electroporated cells are therefore comparable to those found in the 2D culture.

The novelty and advantage of our 3D model is that the cell culture is not in an acqueus medium with a fixed value of conductivity as it happens in 2D models. Rather, we find conductivity conditions more similar to those of the stroma tissue. The similarity between the results of EP in 2D and 3D cultures can confirm that the electric field intensity used could be applied also for an efficient in vivo electroporation. This makes our model more suitable to test the EP efficacy conditions to translate in vivo, since electric fields are applied to a tissue organization closer to human breast cancer.

Moreover, in setting up the electric fields threshold to maximize the transfection efficiency, in 2D model a conductive buffer commonly employed in in vitro transfection protocols has been used. It is important to underline that, due to its toxicity, the buffer cannot be used in in vivo protocols. Therefore, besides the lack of stroma, the 2D model can be quite useful for the general conditions set up but not very predictable for in vivo protocols.

Following this study, we could affirm the scaffold is able to mime the ECM, the cell adhesion to the stroma and the cell–cell junctions that is a more physiological condition, miming what we found in in vivo tumors. Therefore, the use of our in vitro model can represent an important advantage for drug delivery studies based on electrotransfer, since the proposed scaffold could be considered as a new platform for the validation of electroporation conditions to employ in ECT protocols in vivo.

## 5. Conclusions

The range of applications of ECT in cancer treatment is expanding [72,73,74,75]. Despite the high efficacy of the electroporation protocol adopted by the ESOPE guidelines, there remains a need to adapt treatment parameters according to the peculiar tissue characteristics of each tumor type, particularly in bulky cancers where tissue composition may hamper the propagation of electric currents [75]. For these reasons, the investigation of ECT in appropriate settings is advisable. At the same time, the increasing costs of research and several feasibility issues limit the conduction of preclinical or clinical in vivo studies. In this paper, we proposed a new, easy-to-prepare scaffold for 3D cell culture. Notably, after seeding two breast cancer cell lines in the new scaffold, we observed the deposit of EMC around the cells in a way that is similar to in vivo tissues. The primary aim of this study was to propose a new 3D model of cell culture more predictable than the 2D one for the set-up of electroporation conditions. The model proposed could be more useful to translate electrochemotherapy protocols into human cancer tissues.

Interestingly, our 3D model may allow researchers to evaluate drug delivery efficacy particularly in electroporation protocols, in a setting which is, at least for certain aspects, closer to in vivo conditions.
